# Food and Nutrient Intake and Diet Quality among Older Americans

**DOI:** 10.1093/geroni/igaa057.3381

**Published:** 2020-12-16

**Authors:** Yeon Jin Choi, Eileen Crimmins, Jung Ki Kim, Jennifer Ailshire

**Affiliations:** University of Southern California, Los Angeles, California, United States

## Abstract

A suboptimal diet and nutritional deficiencies can have important influences on health with significant impact among older adults. This study aims to assess the presence of suboptimal dietary intake among older Americans and identify risk and protective factors influencing diet quality. For this study, data from a nationally representative sample of 5,614 community-dwelling older adults over age 54 in the Health and Retirement Study – Health Care and Nutrition Survey were used. Descriptive analyses were conducted to assess average intake of 17 food groups and nutrients and the percentage of respondents who consumed an optimal amount of food and nutrients. Differences in diet quality by sociodemographic, psychosocial, environmental, and geographic factors were assessed using chi-square and OLS regression was used to identify risk and protective factors for good quality diet. Overall, only 10.7% of respondents had a good quality diet (HEI score 81 and above); the majority had diets considered poor or needing improvement. Less than 50% of respondents met dietary guidelines and nutritional goals for most individual food groups and nutrients. Respondents with low socioeconomic status, fewer psychosocial resources, and those who had limited access to healthy food outlets were more likely to have a diet of suboptimal quality. Efforts to remove identified barriers that put older adults at risk for poor nutrition and to provide resources that increase access to healthy food should be made to encourage healthy eating and enhance diet quality.

